# Individuals with aphasia generate larger adaptive and corrective responses to suddenly introduced auditory perturbations

**DOI:** 10.3389/fnhum.2025.1672114

**Published:** 2025-11-27

**Authors:** Alexis Basciano, Sara-Ching Chao, Amy Gomez, Ayoub Daliri, Corianne Rogalsky

**Affiliations:** College of Health Solutions, Arizona State University, Tempe, AZ, United States

**Keywords:** aphasia, altered auditory feedback, speech motor control, feedforward control, feedback control

## Abstract

**Introduction:**

Persons with aphasia (PWA) experience changes to feedback and feedforward speech motor control, though the impact on these subsystems has not yet been explored using different perturbation schedules. Here, we examine the magnitude of auditory-motor adaptive and corrective responses together in PWA using gradually and suddenly applied perturbation schedules.

**Methods:**

Nine PWA and 12 neurotypical adults of similar age to the PWA (NT) completed gradual and sudden altered auditory feedback (AAF) paradigms to measure adaptive and corrective responses to formant perturbation (formants of/ε/shifted toward formants of/æ/). As a measure of the feedforward system, we calculated adaptive responses using the formant changes in the first 100 ms of production. As a measure of the feedback system, we calculated corrective responses based on the differences between the later portion of the production (200–300 ms) and the early portion of the vowel (0–100 ms). Our analyses revealed significant interactions between group and perturbation schedule.

**Results:**

Adaptive and corrective responses of PWA were more similar to those of the NT group in the sudden perturbation schedule. Single-case comparisons of each PWA to the NT group identified different impairment patterns in adaptive and corrective responses during gradual and sudden AAF paradigms within PWA.

**Discussion:**

These findings suggest that measuring adaptive and corrective responses using an adaptation paradigm in PWA is both viable and informative, as the PWA exhibited different impairments in adaptive and corrective responses across the two perturbation schedules. Outcome differences in response to sudden versus gradual perturbations for the PWA may be explained by difficulties with the detection of auditory errors during speech. Perhaps a sudden perturbation schedule improves the adaptive and corrective abilities of PWA by increasing their agency over their speech errors. More studies are needed to further elucidate the critical mechanisms of auditory-motor adaptive and corrective responses in PWA.

## Introduction

Interactions between sensory and motor systems are critical during speech development and remain essential throughout the lifespan to produce intelligible speech (Guenther, 2016). Many neurological disorders can significantly impair these interactions, leading to disordered speech (Basilakos and Fridriksson, 2022; Behroozmand et al., 2018; Deger and Ziegler, 2002). Sensorimotor impairments are often present following a brain injury such as a stroke, including in individuals with aphasia (Behroozmand et al., 2018; Behroozmand et al., 2022; Buchsbaum et al., 2011; Sangtian et al., 2021; Johnson et al., 2020). Aphasia is an acquired language disorder impacting an individual’s ability to produce and comprehend language (Damasio, 1992). Because aphasia typically involves damage to left-hemisphere perisylvian regions implicated in sensorimotor speech control (Fridriksson et al., 2018), persons with aphasia (PWA) often show impaired speech production, repetition, and fluency. In addition, there is a high co-occurrence of motor speech disorders in PWA, including acquired apraxia of speech (AOS) (Ziegler et al., 2022). Individuals with AOS exhibit impairments in the planning and programing of speech (Ogar et al., 2005), which significantly impact fluent speech production. However, the link between sensorimotor impairments and disordered speech in PWA is not well understood. Therefore, investigating the sensorimotor system in PWA is crucial for optimizing future treatments to improve speech intelligibility in these patient populations.

Models of speech, including the Directions Into Velocities of Articulators (DIVA) model (Guenther, 2016; Tourville and Guenther, 2011), posit that the speech motor system relies on two subsystems that support these sensory and motor interactions: feedforward and feedback control. The feedforward control system prepares motor commands to produce speech movements and predicts their sensory consequences. The speech motor system compares the sensory prediction with the actual sensory feedback to detect errors in speech output (i.e., prediction errors). The feedforward system uses prediction errors to modify motor commands and thus reduces errors in future productions (e.g., Houde and Jordan, 1998; Villacorta et al., 2007). The feedback system uses the errors to generate corrective responses to reduce errors during the current production (e.g., Purcell and Munhall, 2006; Tourville et al., 2008). This interplay between feedback and feedforward systems enables speech error detection and correction, as well as the optimization of speech motor commands.

Feedback and feedforward subsystems can be examined using altered auditory feedback (AAF) paradigms designed to experimentally induce errors in speech and observe feedforward and feedback systems’ responses to the errors (for a comprehensive review, see Caudrelier and Rochet-Capellan, 2019). When a random, unpredictable perturbation is applied to a participant’s auditory feedback of their own speech, the feedback control system becomes active and generates within-production “*corrective responses*” (also referred to as compensatory responses) opposite to the perturbation to compensate for the induced errors (Purcell and Munhall, 2006; Tourville et al., 2008). If perturbations are repeated over several trials and thus become predictable, the feedforward control system gradually adapts its motor command, in an *adaptive response*, so that future productions compensate for the induced errors by the perturbations (Houde and Jordan, 1998, 2002; Villacorta et al., 2007). These paradigms have been frequently used to study “*adaptive and corrective responses*” in neurotypical adults (Houde and Jordan, 2002; Merrikhi et al., 2025; Munhall et al., 2009; Parrell et al., 2024; Rochet-Capellan and Ostry, 2011) and individuals with Parkinson’s disease (Mollaei et al., 2013), acquired AOS (Ballard et al., 2018), stuttering (Daliri and Max, 2015), and stroke (Behroozmand et al., 2018; Behroozmand et al., 2020; Behroozmand et al., 2022; Behroozmand et al., 2025).

Many PWA (including those with Broca’s aphasia or conduction aphasia) are known to have sensorimotor impairments affecting their speech production and repetition (Behroozmand et al., 2018; Behroozmand et al., 2022; Behroozmand et al., 2025; Buchsbaum et al., 2011; Buchsbaum et al., 2001). Therefore, AAF is a promising tool to both characterize and potentially rehabilitate these sensorimotor impairments in PWA. Previous studies have used randomly applied perturbations to measure changes in corrective responses (i.e., the feedback control system) in PWA (Behroozmand et al., 2018; Behroozmand et al., 2022; Behroozmand et al., 2025; Sangtian et al., 2021). Studies in PWA have found lower magnitude corrective responses compared to those of neurotypical adults of similar age, indicating impairments in the feedback subsystem. Additionally, a study of individuals with both aphasia and AOS identified an impairment of feedforward mechanisms in individuals with both aphasia and acquired AOS, although individuals with aphasia alone were not found to have the same impairments (Maas et al., 2015). However, this particular study was not designed to also characterize feedback impairments. Based on these findings, it is unclear whether PWA are impacted by impairments in the feedback system, feedforward system, or both. A study by Ballard et al. (2018) sought to directly examine adaptive and corrective responses together in PWA using two paradigms: one that gradually applied small incremental errors over time to measure adaptive responses (i.e., examining the feedforward system), and another that randomly applied perturbations to measure corrective responses (i.e., examining the feedback system). Based on their findings, corrective responses within the PWA and neurotypical adults of similar age were as expected; however, neither of these groups exhibited adaptive responses. Importantly, older adults experience age-related changes to their sensory systems, which may have impacted their ability to detect errors (Hu et al., 2023). Thus, the lack of adaptive responses in these groups may be related to the reduced ability of these populations to detect and correct gradually applied perturbations.

There are two prominent paradigms used to characterize adaptive responses experimentally: by introducing errors incrementally over time in a *gradual* adaptation paradigm, or by suddenly introducing and sustaining errors, also known as a *sudden* adaptation paradigm. Studies of limb motor control have used these paradigms to examine various aspects of adaptive responses. For example, studies in the upper limb (perturbations during arm movements) and lower limb (perturbations during walking) have found that adaptive responses to gradual and sudden perturbations are different (Kagerer et al., 1997; Kitatani et al., 2022; van Asseldonk et al., 2011). An upper limb study found different, yet overlapping, brain regions involved in preparing and producing adaptive responses in sudden versus gradual paradigms (Werner et al., 2014). In fact, injuries or diseases influencing the distinct brain regions implicated in producing upper limb adaptive responses to sudden versus gradual paradigms have led to different patterns of responses to suddenly and gradually introduced errors (Criscimagna-Hemminger et al., 2010; Venkatakrishnan et al., 2011). In the speech domain, only a few studies have examined adaptive responses to gradual and sudden paradigms together (e.g., Chao and Daliri, 2023; Kearney et al., 2020; MacDonald et al., 2010; Munhall et al., 2009). The differences between adaptive responses to gradually and suddenly introduced errors have been explained in the context of sense of agency, or an implicit awareness that one is the agent of their actions (Haggard, 2017; Moore, 2016). For example, it has been suggested that the brain may respond more to gradually introduced errors because it may assign smaller errors to its own output rather than external sources (Daliri et al., 2020; Chao and Daliri, 2023). Additionally, neurological disorders such as stroke are known to exhibit impairments in the sense of agency over their movement errors in both the upper limb and speech domains (Miyawaki et al., 2022; Subramaniam et al., 2018). These impairments may influence how the brain internally attributes errors and consequently responds to gradually and suddenly introduced errors. Overall, neurological disorders influence brain regions in different ways, which leads to variation in adaptive responses to gradual and sudden paradigms. This underscores the importance of characterizing responses to gradually and suddenly introduced speech errors in PWA.

It is crucial to examine adaptive and corrective responses to both gradually and suddenly introduced errors to gain a thorough understanding of potential impairments in feedforward and feedback mechanisms in PWA. However, examining adaptive and corrective responses in gradual and sudden paradigms in PWA poses significant practical challenges that must be addressed. PWA often experience cognitive fatigue (Riley, 2017), limiting the feasibility of administering corrective and adaptive response paradigms alongside a full aphasia battery in one session. We recently developed a robust method to efficiently characterize corrective and adaptive responses using a typical adaptation paradigm by examining the early and later portions of a speech segment (Daliri, 2021; Merrikhi et al., 2025). The early portion of a speech segment in a trial (e.g., the first 100 ms) is primarily influenced by the feedforward subsystem, which is represented by the trial-to-trial adaptive changes in speech. The late portion of a speech segment (after 200 ms; e.g., the last 100 ms) is influenced by the feedforward and feedback systems, which are represented by the trial-to-trial adaptive changes and within-trial corrective changes in speech. Thus, subtracting the early response from the late response in a given trial of an adaptation paradigm estimates within-trial corrective responses. Using this approach would enable us to simultaneously and efficiently measure adaptive and corrective responses in PWA within an adaptation paradigm, thereby minimizing the risk of cognitive fatigue in PWA.

The objective of the current study was to characterize the sensitivity of the feedforward and feedback system in PWA to errors that are introduced gradually or suddenly. To address our questions, PWA and neurotypical adults of similar age completed both gradual and sudden AAF paradigms. The adaptation paradigms either gradually or suddenly introduced a formant shift (increase in first formant and decrease in second formant; e.g., “head” would sound like “had”). As a measure of the feedforward system, we used adaptive responses. As a measure of the feedback system, we extracted corrective responses from each trial of the adaptation paradigms (by subtracting changes in formants in the early portion of the vowel from the changes in formants in the later portion of the vowel). We expected that PWA would exhibit different impairments in adaptive and corrective abilities across the perturbation schedules. Specifically, we anticipated improved adaptive and corrective responses in the sudden perturbation schedule in PWA, as the large error induced may surpass their aberrant error-detection threshold (Behroozmand et al., 2018) and/or improve their self-agency over their speech errors (Miyawaki et al., 2022; Subramaniam et al., 2018).

## Materials and methods

### Participants

Nine persons with aphasia (PWA) (Table 1) and 12 right-handed neurotypical adults of similar age (NT) completed the current study. Inclusion criteria for all participants included being over 18 years of age, right-handed (self-reported, right-handed pre-stroke for the PWA group), a fluent monolingual speaker of American English, free of self-reported history of other psychological, neurological, or communication disorders, normal hearing, and no visual impairments (e.g., neglect) that would impede reading single words. Additional PWA criteria included experiencing a stroke at least 6 months prior to participation and a self-reported diagnosis of aphasia [confirmed by the *Boston Diagnostic Aphasia Examination (BDAE) 4th Edition* (Goodglass and Kaplan, 1983)]. No participants exhibited any signs of visual neglect. A GSI 18 Audiometer with supra-aural headphones was used to determine that all participants had a normal hearing threshold for their age in the range of 250–8,000 Hz. Thus, no participants were excluded based on visual or hearing impairment. Participant recruitment occurred through advertisements and word of mouth in the Arizona State University (ASU) community. Neurotypical adult recruitment focused on adults 55 years of age and older, based on the average ages of stroke participants in our previous studies. All participants were compensated hourly for their time. ASU’s Institutional Review Board approved the study.

**TABLE 1:** The table lists demographic, aphasia, and AOS information for the PWA group (*n* = 9).

Subject	Gender	Age	Hearing status (dB)	Months post-stroke	Years education	Aphasia diagnosis	ASRS AOS severity (/4)
SS1	F	69	35.62	130	10	Anomic	0
SS2	M	40	10.83	178	22	Anomic	2
SS3	F	68	30.00	72	18	Anomic	0
SS4	M	59	30.41	69	16	Conduction	0
SS5	M	32	10.41	20	13	Broca’s	0
SS6	F	76	26.00	120	16	Anomic	0
SS7	M	64	39.09	174	14	Broca’s	0
SS8	M	61	35.91	58	16	Anomic	1
SS9	M	83	20.83	114	16	Anomic	0
NTs *n* = 12	8 F	68.17(8.49) Range = (51–79)	26.57 (10.57)	n/a	17.58 (1.88)	n/a	n/a

Aphasia characterization was based on BDAE test scores. Apraxia of speech ratings we scored using the Primary distinguishing features section of the Apraxia Severity Rating Scale (Strand et al., 2014). Only items 1.1 through 1.4 were evaluated from transcribed speech samples, as items 1.5 and 1.6 were difficult to evaluate due to masking at the time of data collection. As in the ASRS, AOS severity was calculated as the average of the symptoms rated (/4), and the total for the symptoms present was the sum out of 16. NT group data comparison presented as mean (SD).

The PWA group was recruited from a shared aphasia registry at ASU laboratories. All PWA were in the chronic stage of recovery (at least 6 months post-stroke, with the group average time since stroke onset being 103 months). Based on performance on the Boston Diagnostic Aphasia Examination (BDAE) (Goodglass and Kaplan, 1983), and following the standard BDAE scoring protocol, the following aphasia types were identified: *n* = 6 anomic aphasia, *n* = 2 Broca’s aphasia, and *n* = 1 conduction aphasia. The BDAE was administered by a trained research assistant.

PWA group demographics were as follows: Age (years) *M* = 61.33, range = 32–83, SD = 16.26; gender = 3 females, 6 males; education (years) *M* = 15.67, SD = 3.3; hearing status (dB HL) *M* = 26.57, SD = 10.57. NT group demographic measures were as follows: Age (years) *M* = 68.17, range = 51–79, SD = 8.49; gender = 8 females, 4 males; education (years) *M* = 17.58, SD = 1.88; hearing status (dB HL) *M* = 29.21, SD = 10.52. *T*-tests revealed no significant differences between NT and PWA groups on age [*t*(19) = -1.25, *p* = 0.226], education [*t*(19) = -1.68, *p* = 0.109], or hearing status [*t*(19) = -0.561, *p* = 0.581]. A chi-squared test revealed no significant association between group and gender, χ^2^(19, *N* = 21) = 2.29, *p* = 0.13.

### Neuropsychological assessments

All tasks were administered by a trained experimenter during a 3-h testing session. All subjects completed a demographic questionnaire. NT mental status was defined using the *Mini Mental Status Exam* (*MMSI*, total score greater than 24) and the *Wechsler Adult Intelligence Scale* (*WAIS*, processing speed and working memory scores within normal range) to exclude any individuals who had cognitive deficits. We administered the *Boston Diagnostic Aphasia Examination (BDAE) 4th Edition* (Goodglass and Kaplan, 1983) to each PWA to confirm an aphasia diagnosis and characterize their aphasia. All PWA in the study demonstrated adequate auditory comprehension skills and single-word reading abilities, which were necessary to follow instructions and complete the experimental tasks. Apraxia of speech (AOS) was qualitatively characterized using select components of the *Apraxia Severity Rating Scale (ASRS)* (see Strand et al., 2014). Not all items from the *ASRS* could be feasibly evaluated at the time of data collection due to the required face mask usage. Thus, AOS severity was assessed with items 1.1 through 1.4 in the *AOS-primary distinguishing features* section of the *ASRS* (*1.1 Distorted sound substitutions, 1.2 Distorted sound additions, 1.3 Increased sound distortions or distorted sound substitutions with increased utterance length or increased syllable/word articulatory complexity, 1.4 Increased sound distortions or distorted sound substitutions with increased speech rate*). Each AOS feature in the scale was rated on a scale from 0 to 4, with 0 indicating the speech characteristic was not present in the sample and four being the characteristic was prominent and severe throughout the sample. As in the *ASRS*, AOS severity was calculated as the average of the symptoms rated (/4), and the total for the symptoms present was the sum out of 16. Due to incomplete characterization of AOS via a partial *ASRS*, the partial *ASRS* scores were only used to qualitatively describe speech characteristics and were not used as regressors in analyses.

### Altered auditory feedback task design

**FIGURE 1:**
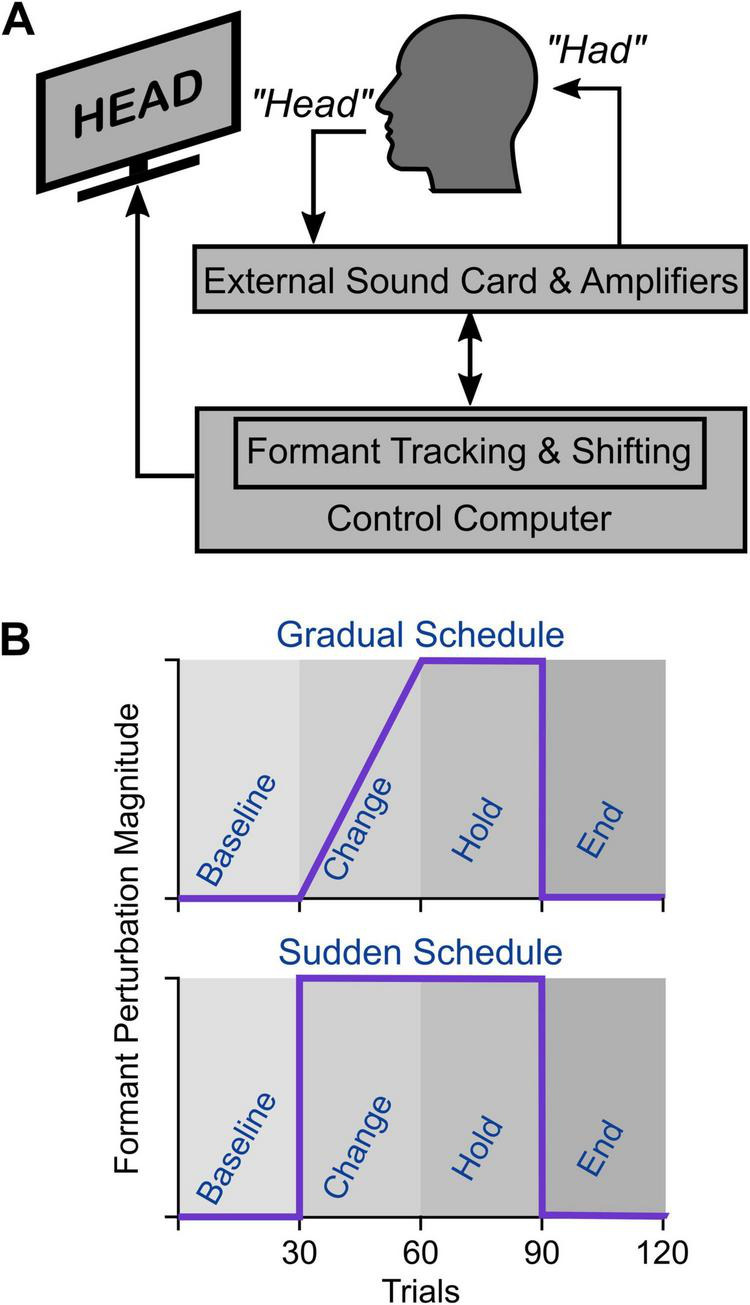
**(A)** The experimental AAF apparatus. **(B)** The perturbation schedule for the gradual adaptation paradigm: 30 baseline trials with no perturbation, followed by 30 trials of the ramp phase, gradually increasing the perturbation up to maximum, followed by 30 trials of the hold phase at maximum perturbation, followed by 30 trials of after-effects with no perturbation applied. Perturbation schedule for the sudden adaptation paradigm: 30 baseline trials with no perturbation, followed by 60 trials of maximum perturbation, followed by 30 trials of after effects with no perturbation applied.

The altered auditory feedback (AAF) experimental task and laboratory setup (Figure 1) have been extensively described previously (Daliri, 2021; Daliri et al., 2020). Briefly, the experiment was conducted in a sound booth where participants sat five feet from a 24-inch computer monitor wearing insert headphones (ER-2, Etymotic Research Inc.). Speech signals were collected using a mounted microphone (SM58, Shure) positioned at a 45-degree angle approximately 15 cm from the participant’s mouth. The microphone signal was amplified (Tubeopto 8, ART Pro Audio) and sampled at 48,000 Hz using an external audio interface (8Pre USB, Motu). The output of the audio interface was then amplified (Eurorack, Behringer) and played back to the participant via insert earphones. The external audio interface was connected to a computer that was used to record speech samples and control various aspects of the experiment. We used Audapter (Cai, 2015) to estimate and manipulate formant frequencies in near real-time (with ∼18 ms input-to-output delay). Prior to the testing session, the experimental setup was calibrated so the insert headphones output signal was 5 dB higher than the input signal from the microphone.

All participants completed the following: a practice task, a pre-test task, and two adaptation tasks (sudden and gradual adaptation). At the start of each trial throughout each of the tasks, a monosyllabic word was displayed on the screen for 3 s. Participants were instructed to say the displayed word while maintaining constant speech intensity and prolonging their vowels. The goal of the practice task was to familiarize participants with the experimental setup and to produce speech while hearing themselves in the insert earphones. The practice task included 24 repetitions of /ε/ in the context of monosyllabic words (“hep,” “head,” and “heck”) under normal auditory feedback. During this task, participants received visual feedback on their speech intensity and duration, enabling them to maintain optimal speech quality for subsequent experiments. All participants were able to complete the task successfully after 1–3 repetitions of the practice task. Following the practice task, participants completed a pre-test task. The goal of the pre-test task was to determine participant-specific vowel configurations that were used to develop participant-specific formant perturbations. During this task, participants produced 25 repetitions of /i/, /ε/, and /æ/ in the context of /hVp/. Immediately after the completion of this task, we used estimated formant trajectories by Audapter to calculate the average formant values for each production. We then used these averaged formant values to determine the participant-specific vowel centroid for each of the vowels /i/, /ε/, and /æ/. We visually inspected the calculated vowel centroids and averaged formant values for each participant to ensure the quality of the vowel centroids. We used these vowel centroids as initial values for formant tracking and to define participant-specific formant shifts in subsequent adaptation tasks.

After the pre-test task, participants completed two blocks of the adaptation task with gradual or sudden introduction of formant shifts (Figure 1B). In each block, participants produced 120 repetitions of the vowel /ε/ in the context of a monosyllabic word (“hep,” head,” or “heck”). We shifted the first formant (F1) and the second formant (F2) such that the formants of the vowel /ε/ for a participant were shifted toward the formants of the vowel /æ/ (i.e., F1 increased and F2 decreased). The magnitude and direction of formant shift were calculated using the participant-specific centroids of /ε/ and /ae/ (e.g., “head” would sound like “had”). The gradual adaptation paradigm contained 30 baseline trials (with no perturbation), 30 ramp trials (with gradual perturbations up to maximum magnitude), 30 hold trials (with maintained perturbation at maximum magnitude), and 30 after-effect trials (with no alterations). The sudden adaptation task was identical to the gradual task; only there was a full formant perturbation shift from trials 31–60 in place of a gradual shift (Figure 1B). The administration order of the gradual and sudden adaptation tasks was counterbalanced to control for potential order effects. Participants completed a simple sentence reading task with no speech alteration between adaptation tasks to minimize carryover effect from one adaptation task to the next adaptation task.

### Speech processing and analysis

A custom-written MATLAB script was used to visually inspect and manually extract target vowel productions from each utterance. Production errors (such as mistakes) were monitored and excluded in this stage. Once the vowel onsets and offsets were isolated, MATLAB scripts were used to extract formant frequencies from each production automatically. Average formant values in the early time window (0–100 ms of each production) contained the adaptive response, and average formants in the late time window (200–300 ms of each production) contained both adaptive and corrective responses. Early response was subtracted from late response to estimate corrective responses (Daliri, 2021). Average responses in the baseline trials were used to normalize productions in the remaining phases for both early and late responses. Data was split into target words (head, heck, and hep) to account for coarticulation effects due to formant transition induced by the ending consonants.

### Statistical analyses

Average baseline corrected responses during the hold phase of each paradigm (measured by changes in F1 and F2 adaptive and corrective responses in Hz) were used as the dependent variables for statistical analyses. The hold phase was the primary focus of our analyses because it is the phase in which adaptive response magnitude is most likely to be the greatest (Houde and Jordan, 2002), and therefore of particular interest to our examination of the viability of measuring adaptive responses in PWA. Statistical analyses were carried out using R programing version 4.1.1 (R Core Team, 2021). The dependent variable for all participants was the change in Hz during 30 hold phase trials during each paradigm. Four linear mixed effects models were fitted to the dataset to determine statistical significance using the R lmer package (Kuznetsova et al., 2017) with group and paradigm (sudden and gradual) as the fixed factors. The R emmeans package (Lenth, 2023) was used to explore *post hoc* interactions using the Kenward-Roger method (Halekoh and Højsgaard, 2014) to calculate degrees of freedom and the Sidak method (Sidak, 1967) to correct for multiple comparisons. IBM SPSS Statistics (Version 28) was used to further explore relationships between cognitive assessment performance and adaptive and corrective responses. To explore the heterogeneity of the PWA group, the DissocsBayes_ES.exe open-source computer program (Crawford and Garthwaite, 2005) was used to determine whether individual PWA had an impairment in task performance compared to the NT group. For brevity, these analyses were focused on the change in Hz for F1. Here, the average hold phase F1 Hz change for adaptive and corrective responses in each paradigm (sudden, gradual) for each PWA was compared to the average, standard deviation, and correlation of the tasks in the NT group. The open-source program indicated whether or not the PWA had an impairment in either task, with impairment defined as a probability of < 0.05 that the PWA’s performance is an observation from the NT group using Crawford and Garthwaite’s (2005) test.

## Results

### Adaptive and corrective responses

**FIGURE 2:**
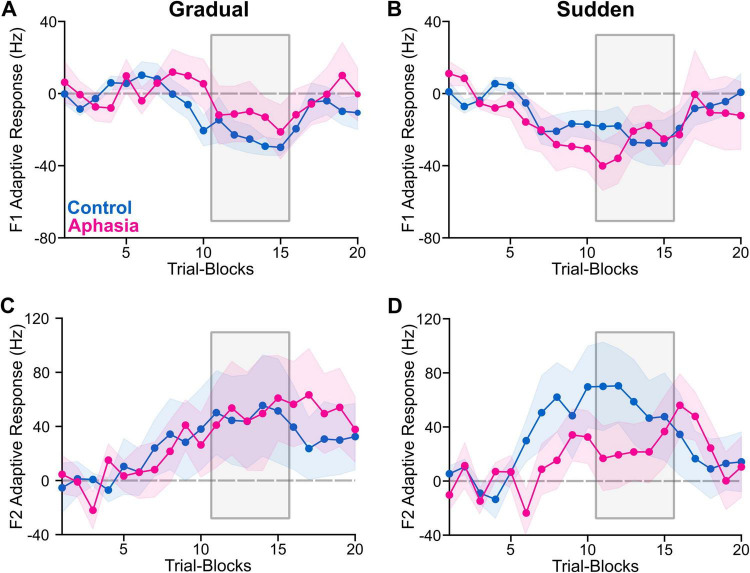
The figure shows group average adaptive response for NTs (blue) and PWA (pink), indicated by plotted lines and variability represented by corresponding shading. The phase of interest (hold) is indicated by the gray box. The perturbation increased F1 and decreased F2; therefore, the expected adaptive response is negative for F1 and positive for F2. For visual presentation, we averaged the adaptive responses from each 120-trial task were averaged in bins of six consecutive trials, yielding 20 data points per task. The color-shaded areas are the standard errors of the mean. **(A)** Average F1 adaptive response (Hz) for the gradual task. **(B)** Average F1 adaptive response for the sudden task. **(C)** Average F2 adaptive response for the gradual task. **(D)** Average F2 adaptive response for the sudden task.

**FIGURE 3:**
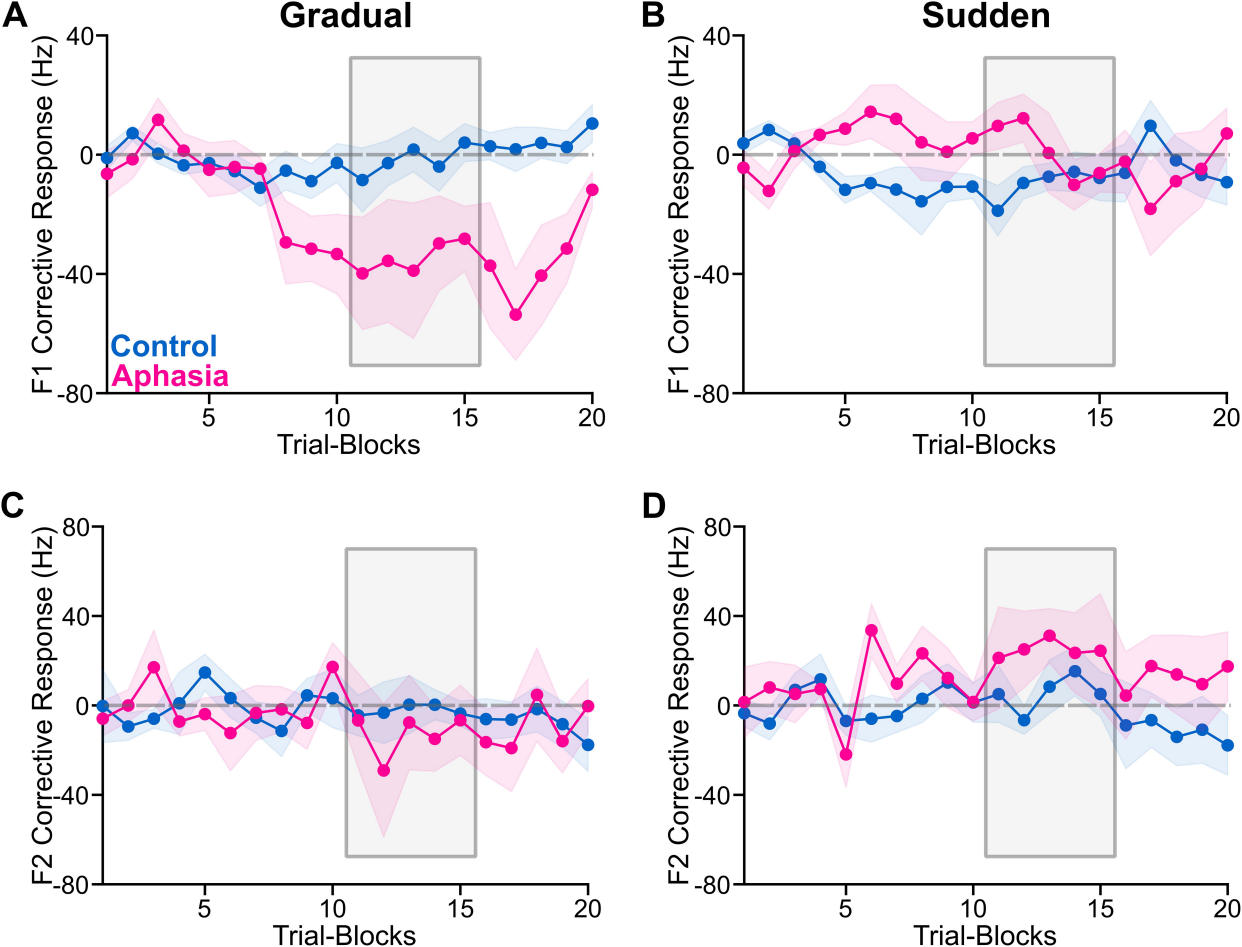
The figure shows group average corrective response for NTs (blue) and PWA (pink), indicated by plotted lines and variability represented by corresponding shading. The phase of interest (hold) is indicated by the gray box. The perturbation increased F1 and decreased F2; therefore, the expected corrective response is negative for F1 and positive for F2. For visual presentation, we averaged the corrective responses from each 120-trial task were averaged in bins of six consecutive trials, yielding 20 data points per task. The color-shaded areas are the standard errors of the mean. **(A)** Average F1 corrective response (Hz) for the gradual task. **(B)** Average F1 corrective response for the sudden task. **(C)** Average F2 corrective response for the gradual task. **(D)** Average F2 corrective response for the sudden task.

**FIGURE 4:**
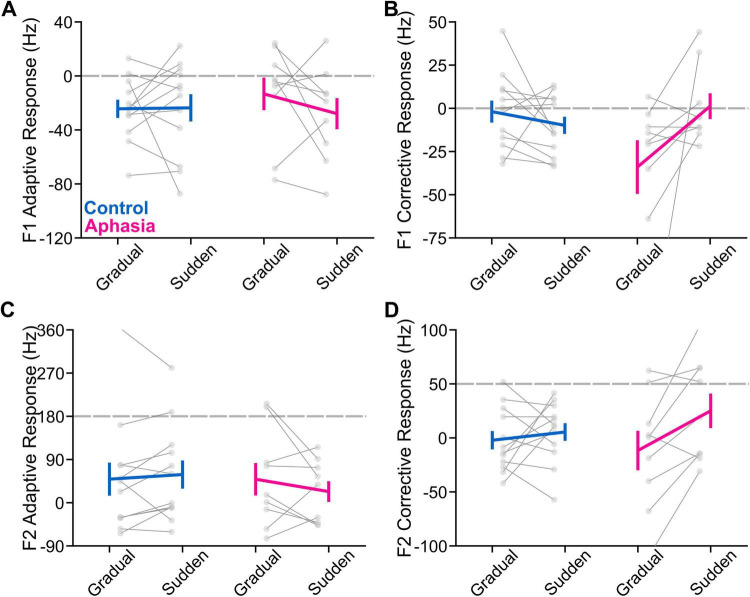
The figure shows group average adaptive and corrective responses (NTs in blue and PWA in pink) along with individual performance differences for each measure in gray. The error bars are the standard errors of the mean. **(A)** Gradual and sudden task F1 adaptive response. **(B)** Gradual and sudden F1 corrective response. **(C)** Gradual and sudden F2 adaptive response. **(D)** Gradual and sudden F2 corrective response.

**FIGURE 5:**
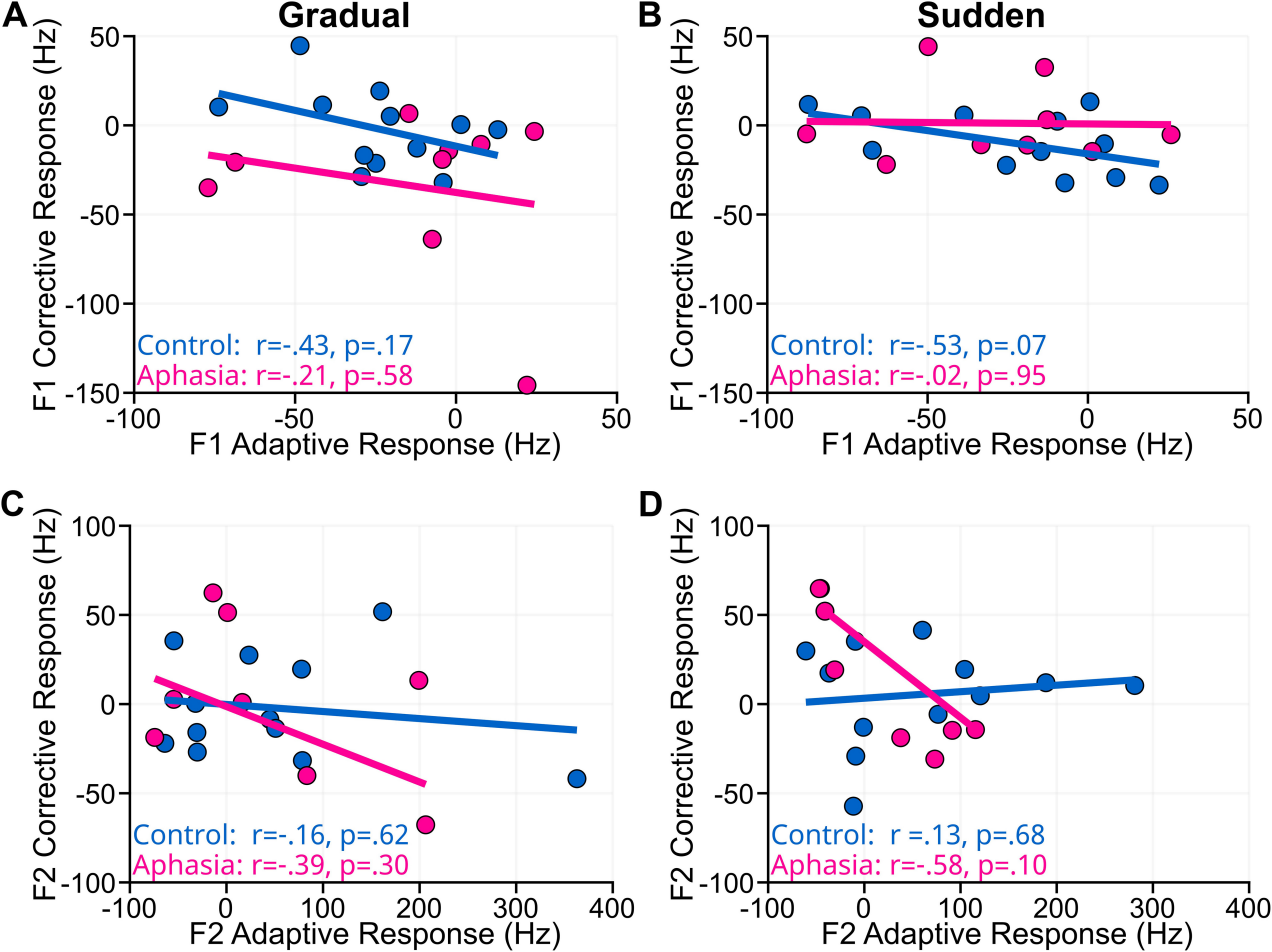
The figure shows plotted correlations between corrective and adaptive responses for gradual and sudden paradigms for PWA (red) and NT (blue). All correlations between corrective and adaptive responses for both groups across paradigms were not statistically significant for F1 and F2. **(A)** F1 adaptive versus corrective responses for the gradual task. **(B)** F1 adaptive versus corrective for the sudden task. **(C)** F2 adaptive versus corrective response for the gradual task. **(D)** F2 adaptive versus corrective response for the sudden task.

F1 and F2 adaptive and corrective responses (in Hz) in the hold phase for each of the PWA and the group-average of the NT group are reported in Tables 2, 3. Group average adaptive and corrective responses (along with variability) for each formant are depicted in Figures 2, 3 (for visualization, adaptative responses from each 120-trial task were averaged in bins of six consecutive trials, yielding 20 data points per task). Group (and individual) average adaptive and corrective responses for gradual and sudden paradigms for each formant are depicted in Figure 4. The results of the four linear mixed models are reported in Table 4. Notably, there was no significant main effect of group in any of the models. There was a significant paradigm main effect for F1 adaptive response [*F*(1, 1,007) = 12.58, *p* < 0.001], F1 corrective response [*F*(1, 946) = 34.81, *p* < 0.001), and F2 corrective response [*F*(1, 966) = 22.54, *p* < 0.001]. All group*paradigm main effects were found to be significant: F1 adaptive: [*F*(1, 1,007) = 20.28, *p* < 0.001], F2 adaptive [*F*(1, 1,026) = 16.10, *p* < 0.001], F1 corrective [*F*(1, 946) = 57.25, *p* < 0.001], F2 corrective [*F*(1, 966) = 23.67, *p* < 0.001]. *Post hoc* contrasts (Table 5 and Figure 5) demonstrated that adaptive and corrective responses for both F1 and F2 were significantly different between gradual and sudden within the aphasia group (all *p* < 0.05). This significant paradigm effect was not found in the NT group, for F1 adaptive response [*t*(1,007) = -0.78, *p* > 0.05], F1 corrective response [*t*(944) = 1.4, *p* > 0.05], or F2 corrective response [*t*(965) = 0.096, *p* > 0.05], though it was significant for F2 adaptive response [*t*(1,026) = -2.7, *p* < 0.05].

**TABLE 2:** The table lists the average change (in Hz) for F1 adaptive and corrective responses in each paradigm.

Subject	Sudden		Gradual	
	Adaptive	Corrective	Adaptive	Corrective
SS1	−18.91	−11.10	7.76	−10.68
SS2	−33.32	−10.90	−68.58	−20.68
SS3	−62.86	−21.95	−2.29	−14.19
SS4	25.93	−5.26	−14.63	6.65
SS5	−87.73	−4.78	−77.02	−35.00
SS6	−13.53	32.47	22.03	−145.74
SS7	−12.77	3.13	−4.26	−19.13
SS8	−49.83	44.14	24.33	−3.43
SS9	1.36	−14.66	−7.40	−63.88
PWA group (*n* = 9)	−27.96(34.61)	1.23(22.33)	−13.34(36.17)	−34.01(46.50)
NT group (*n* = 12)	−23.66(35.02)	−9.85(17.22)	−24.36(23.35)	−1.92(22.03)

Responses were baseline corrected and averaged over the thirty trials of the hold phase for each participant (denoted by SS, stroke subject). PWA and NT group averages and standard deviations for each measure are presented as mean (SD).

**TABLE 3:** The table lists the average change (in Hz) for F2 adaptive and corrective responses in each paradigm.

Subject	Sudden		Gradual	
	Adaptive	Corrective	Adaptive	Corrective
SS1	−30.79	19.18	−74.27	−18.69
SS2	91.45	−14.72	206.29	−67.71
SS3	−45.64	64.84	16.32	0.89
SS4	115.52	−14.31	83.29	−40.07
SS5	54.65	103.60	199.19	13.29
SS6	73.27	−30.86	76.72	−109.58
SS7	−46.86	64.75	1.11	51.35
SS8	37.84	−18.87	−54.63	2.65
SS9	−40.96	52.10	−13.94	62.36
PWA group (*n* = 9)	23.16(64.78)	25.08(47.80)	48.90(101.55)	−11.72(54.80)
NT group (*n* = 12)	58.63(101.64)	5.43(28.25)	49.07(119.18)	−2.16(29.38)

Responses were baseline corrected and averaged over the thirty trials in the hold phase for each participant (denoted by SS, stroke subject). PWA and NT group averages and standard deviations for each measure are presented as mean (SD).

**TABLE 4:** The table lists the F-statistics and *p*-values for each factor.

	Adaptive		Corrective	
Factors	F1	F2	F1	F2
Group	*F*(1, 18.77) = 0.01, *p* = 0.898	*F*(1, 19.03) = 0.05, *p* = 0.821	*F*(1, 19.6) = 2.10, *p* = 0.162	*F*(1, 18.9) = 0.10, *p* = 0.753
Paradigm	***F*(1, 1,007) = 12.58, *p* < 0.001**	*F*(1, 1,026) = 0.43, *p* = 0.510	***F*(1, 946) = 34.81, *p* < 0.001**	***F*(1, 966) = 22.54, *p* < 0.001**
Group*paradigm	***F*(1, 1,007) = 20.28, *p* < 0.001**	***F*(1, 1,026) = 16.10, *p* < 0.001**	***F*(1, 946) = 57.25, *p* < 0.001**	***F*(1, 966) = 23.67, *p* < 0.001**

Significant findings are bolded.

**TABLE 5:** The table lists the t-statistics and *p*-values for *post hoc* analysis contrast results.

	Adaptive		Corrective	
Contrast	F1	F2	F1	F2
PWAgradual-NTgradual	*t*(21) = 1.2, *p* > 0.05	*t*(19.3) = 0.13, *p* > 0.05	***t*(24.6) = −3.9, *p* < 0.05**	*t*(21.8) = −1.5, *p* > 0.05
PWAgradual-PWAsudden	***t*(1,008) = 5.1, *p* < 0.05**	***t*(1,026) = 3.0, *p* < 0.05**	***t*(946) = −8.5, *p* < 0.05**	***t*(967) = −6.1, *p* < 0.05**
NTgradual-NTsudden	*t*(1,007) = −0.78, *p* > 0.05	***t*(1,026) = −2.7, *p* < 0.05**	*t*(944) = 1.4, *p* > 0.05	*t*(965) = 0.096, *p* > 0.05
PWAsudden-NTsudden	*t*(20.7) = −0.81, *p* > 0.05	*t*(19.3) = −0.54, *p* > 0.05	*t*(24.1) = 1.2, *p* > 0.05	*t*(21.4) = 0.89, *p* > 0.05

Significant findings are bolded.

### Correlations analysis

Figures 5A–D depict correlations between adaptive and corrective responses for sudden and gradual paradigms. NT group F1 adaptive and corrective responses were not significantly correlated for the gradual paradigm (*r* = -0.43, *p* = 0.17) or the sudden paradigm (*r* = -0.53, *p* = 0.07). The NT group F2 adaptive and corrective responses were not significantly correlated for F2 sudden (*r* = 0.13, *p* = 0.68) or F2 gradual (*r* = 0.16, *p* = 0.62) paradigms. No significant correlations between adaptive and corrective responses were found for the PWA group: F1 gradual (*r* = -0.21, *p* = 0.58), F1 sudden (*r* = -0.02, *p* = 0.95), F2 gradual (*r* = -0.39, *p* = 0.30), and F2 sudden (*r* = -0.58, *p* = 0.10) paradigms. Performance on *WAIS* or *BDAE* subtests was not significantly correlated with performance in either paradigm within the PWA group. For the NT group, sudden F1 adaptive responses were significantly correlated with scaled *WAIS* Digit-span scores (*r* = 0.72, *p* = 0.008). The correlation between gradual and sudden adaptive responses in F2 for NTs was significant (*r* = 0.75, *p* = 0.005). *WAIS* Digit-span scores were also correlated with gradual F2 adaptive (*r* = -0.62, *p* = 0.033) and sudden F2 adaptive response (*r* = -0.76, *p* = 0.004). All other NT correlations were non-significant.

### Single case comparisons

To identify individual PWA with significant deficits on adaptive and corrective responses compared to the NT group, single-case Bayesian *t*-tests were computed to identify PWA demonstrating an impairment on each measure, with impairment defined as a probability of < 0.05 that the PWA’s performance is an observation from the NT group (Crawford and Garthwaite, 2005). The results (Tables 6, 7) indicate a variety of patterns of adaptive and corrective impairment across the gradual and sudden paradigms, indicating individual variability in the relationship between adaptive and corrective responses in PWA, and in the effects of gradual and sudden schedules on these measures. Four PWA (notably with three different types of aphasia) did not exhibit any impairments (SS1, SS3, anomic; SS4, conduction; SS7, Broca’s). The other five PWA exhibited impairment in at least one measure in one paradigm schedule compared to the control group: SS2 (anomic) exhibited significantly larger adaptive response in the gradual paradigm (*t* = -1.82, *p* = 0.048); SS5 (Broca’s) exhibited significantly larger adaptive response in both the sudden (*t* = -1.76, *p* = 0.053) and gradual *t* = -2.17, *p* = 0.027 paradigms; SS6 (anomic) exhibited significantly smaller adaptive response *t* = 1.91, *p* = 0.041) and larger corrective response (*t* = -6.27, *p* = 0.001) in the gradual paradigm, but smaller corrective response in the sudden paradigm *t* = 2.36, *p* = 0.019; SS8 (anomic) exhibited significantly smaller adaptive response in the gradual paradigm (*t* = 2.00, *p* = 0.0353, and smaller corrective response in the sudden *t* = 3.01, *p* = 0.006); SS9 (anomic) exhibited significantly larger corrective response in the gradual paradigm (*t* = -2.70, *p* = 0.010).

**TABLE 6:** Deficit significance results are listed as (two-tailed t statistic, *p*-value), and represent how different the individual’s score is compared to the NT group.

	Gradual		Sudden	
Subject	Adaptive	Corrective	Adaptive	Corrective
SS1	1.32, 0.107	−0.38, 0.355	0.13, 0.449	−0.07, 0.473
SS2	−**1.82, 0.048**	−0.82, 0.215	−0.27, 0.398	−0.06, 0.477
SS3	0.91, 0.192	−0.54, 0.301	−1.08, 0.153	−0.68, 0.257
SS4	0.40, 0.348	0.37, 0.358	1.36, 0.101	0.26, 0.401
SS5	−**2.17, 0.027**	−1.44, 0.088	−**1.76, 0.053**	0.28, 0.391
SS6	**1.91, 0.041**	−**6.27, 0.001**	0.28, 0.393	**2.36, 0.019**
SS7	0.83, 0.213	−0.75, 0.234	0.30, 0.385	0.72, 0.242
SS8	**2.00, 0.035**	−0.07, 0.474	−0.72, 0.244	**3.01, 0.006**
SS9	0.70, 0.250	−**2.70, 0.010**	0.69, 0.253	−0.27, 0.397

SS represents stroke subject. Significant values are bolded.

**TABLE 7:** The table lists impaired and unimpaired F1 gradual adaptive and corrective responses for individual PWA.

Impairment category	Number of PWA participants
Impairment in adaptive response only	3
Impairment in corrective response only	1
Impairment in both	1
No impairment	4

## Discussion

Sensorimotor interactions between feedforward and feedback systems are crucial to produce intelligible speech and to correct for error (Guenther, 2016). These interactions are frequently impaired in individuals with acquired disorders such as aphasia and AOS (Basilakos and Fridriksson, 2022). AAF is a behavioral paradigm that has been used in these clinical populations to characterize their feedforward impairments, via adaptive responses to sustained perturbation (Houde and Jordan, 2002), and feedback impairments, via corrective responses to unpredictable perturbation (Tourville et al., 2008). Previous studies using AAF in PWA (Behroozmand et al., 2018; Behroozmand et al., 2022) and AOS (Ballard et al., 2018; Maas et al., 2015) have identified different impairment characteristics in adaptive and corrective responses. These findings may be due to the nature of the gradual adaptation paradigms used in these studies, where the perturbation applied to their speech is too small to be detected by individuals with impairments in error detection (Behroozmand et al., 2018). An alternative paradigm, sudden adaptation, introduces a large error suddenly, which may lead to different effects in these populations (Kearney et al., 2020; MacDonald et al., 2010; Munhall et al., 2009). However, adaptive and corrective responses to both gradual and sudden AAF paradigms had not yet been compared directly in PWA. Thus, in the current study, nine PWA and 12 neurotypical adults of similar age completed two AAF adaptation paradigms, with gradual and sudden perturbation schedules to (1) directly compare the performance of feedforward and feedback systems in PWA via measures of adaptive and corrective responses during AAF and (2) determine whether using different perturbation schedules impacts these responses in the PWA group. We hypothesized that PWA would have improved adaptive and corrective responses in the sudden paradigm, as the large error applied would surpass their aberrant error detection threshold and increase their agency over their speech errors.

To test our hypothesis, we compared baseline corrected adaptive and corrective responses (change in Hz in F1 and F2) across the gradual and sudden paradigms. To mitigate cognitive fatigue within the PWA group and maximize efficiency in data collection, corrective responses were extracted from adaptive responses by subtracting early responses from late responses for each trial. Based on our analyses of F1 adaptive, F2 adaptive, F1 corrective, and F2 corrective responses, group differences in adaptive and corrective responses across paradigms between PWA and NT groups were not significant, likely due to a range of aphasia types and severity. However, there was a significant interaction between group and paradigm. In support of our hypothesis, *post hoc* analyses indicate that both adaptive and corrective responses were greater for the PWA group in the sudden perturbation paradigm compared to the gradual. These behavioral differences may be explained by differences in sensory feedback reliability in PWA. In neurotypical individuals, the reliability and relevance of the sensory feedback impact the magnitude of correction in response to the error (Daliri and Dittman, 2019; Niziolek and Parrell, 2021). Sensory feedback that is too far from the target is more likely to be considered irrelevant and to be externally attributed, while smaller errors in sensory feedback lead to larger changes in behavior in neurotypical participants (Chao and Daliri, 2023; Daliri et al., 2020; Daliri and Dittman, 2019). Previous studies using AAF have found PWA may exhibit lower magnitude auditory-motor correction due to deficits in auditory-error feedback detection (Behroozmand et al., 2018; Behroozmand et al., 2022; Johnson et al., 2020). The unreliability of sensory feedback can impact error attribution, which in turn can affect adaptive and corrective responses.

An alternative interpretation of these findings is in the context of agency (Haggard, 2017; Moore, 2016). The extent to which an individual corrects for error is thought to be connected to a sense of agency, or an awareness of oneself having control over their actions (Subramaniam et al., 2018). Once the brain calculates error, it evaluates the source of the error. If errors are large, unreliable, or ambiguous (Couchman et al., 2012) the brain may attribute them to external sources rather than its own input, leading to a reduced corrective response. However, if the errors are small and reliable, the brain is more likely to attribute the error source implicitly and thus apply corrections (Chao and Daliri, 2023; Daliri et al., 2020). This implies a relationship between agency and error-source assignment in speech (Couchman et al., 2012; Franken et al., 2022). Agency can be broken down into implicit agency (an objective feeling of agency) and explicit agency (a subjective awareness of agency) (Synofzik et al., 2008). These components are thought to be supported by separate neural networks (Applebaum et al., 2025; Moore et al., 2012) with the implicit component driven largely by lower-level comparator processes (Frith et al., 2000), and the explicit component driven by higher-level decision-making processes. In PWA, who often have damage to sensorimotor networks, a sudden perturbation schedule may improve adaptive responses by engaging these decision-making processes and consequently improving the explicit detection of speech errors. However, these are speculations and future studies with lesion data and larger sample sizes would be needed to draw these conclusions. Interestingly, our findings in the sudden paradigm contrast the literature in limb rehabilitation paradigms for stroke, which have found better rehabilitation when using a gradual paradigm (Reisman et al., 2007; Sawers et al., 2013; Park et al., 2021). These differences likely reflect the inherent distinctions between auditory and visual sensory modalities (Grimme et al., 2011).

Single case statistics revealed significant deficits in adaptive and corrective responses in five of the nine PWA. Like previous studies, some individuals had significant impairments in corrective responses, likely due to impairments of feedback error detection (Behroozmand et al., 2018, 2022; Johnson et al., 2020). However, these impairments did not consistently extend to the feedforward subsystem, as reflected in adaptive responses. Within the three PWA who exhibited impairments in adaptive responses only, two of those individuals exhibited qualitative features of AOS (SS2 and SS8). This finding supports the notion of feedforward subsystem impairments underlying speech characteristics in AOS (Maas et al., 2015; Terband et al., 2020). It may be the case that PWA who are also affected by AOS are more likely to present with feedforward system impairments. However, a larger PWA/AOS sample with more detailed, quantitative characterization of AOS is needed to confirm this. Overall, in the current study, different PWA had different impairments in adaptive and corrective responses. Therefore, examining both types of responses is needed for a complete picture of auditory-motor deficits in PWA. While these findings of differences between adaptive and corrective responses suggest that feedback and feedforward subsystems are supported by different neurological networks, our sample size is small. Therefore, future neuroimaging studies with larger aphasia samples are needed to confirm.

Examining adaptive and corrective responses using different perturbation schedules in PWA is a promising direction for the field, as it has the potential to elucidate critical mechanisms of neural speech motor control. Based on our results, AAF paradigms are a viable method to measure both auditory-motor adaptive and corrective abilities in PWA. Individuals with various aphasia and AOS profiles were all able to successfully complete both the sudden and gradual adaptation paradigms. While the results of the current study are the first to indicate PWA adaptation and corrective responses to AAF were improved using a sudden perturbation schedule, future studies of the relationship between auditory-motor integration and aphasia are needed to fully understand the therapeutic implications of these findings.

### Limitations

Future studies should incorporate larger sample sizes to provide sufficient power to elucidate differences in auditory-motor integration abilities within all classifications and severity levels of aphasia. We expect there to be a link between aphasia type and sensorimotor impairment. For example, we would expect that individuals with Broca’s aphasia would be more likely to have a feedforward impairment due to damage to frontal speech control regions, and that individuals with conduction aphasia would be more likely to have a feedback impairment due to damage to posterior temporal auditory-motor integration regions (see Behroozmand et al., 2018). However, the PWA sample size in the current study was not large enough to draw these conclusions. Therefore, future studies with larger and more diverse aphasia samples could potentially address these important questions related to specific impairments relative to aphasia type.

Some PWA in our sample showed feedforward impairments, reflected in reduced adaptive responses, though our behavioral measures do not identify their underlying cause. One way to further characterize these impairments is to examine how sensory systems in PWA are engaged during speech planning. During speech planning, the brain normally prepares auditory cortices for expected sensory outcomes of movement (Guenther, 2016). This has been observed experimentally as a reduced auditory N1 event-related potential (ERP) response to a tone presented during speech planning compared to passive listening—a phenomenon known as prespeech auditory modulation (PSAM) (Daliri and Max, 2018). Although the precise role of PSAM is not fully understood, reduced modulation during planning has been linked to speech impairments (Daliri and Max, 2018). Thus, a lack of PSAM in PWA may underlie their feedforward impairments. Testing this hypothesis would require future studies directly comparing PSAM to adaptive and corrective responses. This approach could clarify the nature of feedforward impairments and point toward strategies for enhancing sensory involvement during speech planning in PWA.

While the heterogeneous adaptive and corrective responses observed in the PWA group suggest these processes have distinct neuroanatomical correlates, lesion data are needed in future studies to directly test this hypothesis. Targeted stimulation studies have implicated the ventral motor cortex as of critical importance for updating future motor commands (Behroozmand et al., 2020; Scott et al., 2020). Lesions to this area in PWA could critically affect the feedforward system, impairing their ability to update future motor commands, regardless of prediction error or perturbation schedule. Additionally, the effects of aging on sudden versus gradual perturbation performance need to be further characterized. Our neurotypical group was matched in age to our PWA group, with a mean age of 68 years. Older adults are known to rely more on feedforward predictions, as their sensory feedback may become less reliable due to age-related changes (Hu et al., 2023; Wolpe et al., 2016). Thus, a more holistic understanding of how the auditory-motor system changes with age is needed to fully understand the changes observed in adults of all ages with aphasia.

Lastly, we acknowledge that observed differences in performance between gradual and sudden paradigms in the PWA group could be driven by alternative differences between the two tasks. Specifically, the sudden paradigm contained more trials with maximum perturbation (60 total trials) compared to the gradual paradigm (30 total trials). However, a recent AAF adaptation study by Kim et al. (2025) found that adaptive responses in speech are more influenced by the amount of time of exposure to the perturbation as opposed to the number of trials containing the perturbation. Given that the gradual and sudden paradigms took the same amount of time to administer in our study, we do not expect that the increased number of maximally perturbed trials in the sudden paradigm to be the driving factor in our findings.

## Conclusion

For the first time to our knowledge, adaptive and corrective abilities in PWA and neurotypical adults of similar age were examined using AAF sudden and gradual perturbation schedules. We found that using AAF adaptation paradigms is a viable method to investigate feedforward (and feedback) subsystems in aphasia. An understanding of both adaptive and corrective abilities is critical to completely characterize individual deficits in motor speech control, as we found that these abilities can differ in PWA. Additionally, the sudden perturbation schedule yielded adaptive and corrective responses in PWA closer to those of the NT group. These differences in sudden versus gradual perturbations may be explained by the sudden perturbation changing auditory-error gain (i.e., increasing sensitivity to errors in their auditory feedback), by influencing agency and/or awareness of speech errors in PWA. Investigating the integrity of feedback and feedforward subsystems in PWA is a promising means to elucidate the critical mechanisms of speech motor control and to guide targeted neural and behavioral treatments for PWA.

## Data Availability

The raw data supporting the conclusions of this article will be made available by the authors, without undue reservation.
